# Blood transcript analysis and metastatic recurrent small bowel carcinoid management

**DOI:** 10.1186/1471-2407-14-564

**Published:** 2014-08-05

**Authors:** Irvin M Modlin, Ignat Drozdov, Lisa Bodei, Mark Kidd

**Affiliations:** Wren Laboratories, 35 NE Industrial Road, Branford, CT 06405 USA; Bering Limited, Richmond, UK; Division of Nuclear Medicine, European Institute of Oncology, Milan, Italy; Department of Surgery, Yale University School of Medicine, New Haven, CT 06510 USA

**Keywords:** Biomarker, Blood, Carcinoid, Chromogranin A, ^68^Gallium, Gene marker, Neuroendocrine tumor, PCR, PET/CT

## Abstract

**Background:**

Detection of neuroendocrine tumor (NET) disease progression is a key issue in determining management. Currently, assessment is by imaging (MRI/CT and Octreoscan®) and plasma Chromogranin A (CgA) measurement.

**Case presentation:**

We report use of a NET-specific multigene PCR-derived blood transcript signature (NET Index) to assess disease and correlated CgA and gene transcripts with MRI, CT, Octreoscan®, ^11^C-5HTP-PET/CT and ^68^Ga-DOTA-PET/CT in a patient with NET.

**Conclusions:**

Our results identify limitations in evaluating disease status by CgA and identify that a PCR-based test is more sensitive. Alteration in NET blood gene transcript levels prior to image-based tumor confirmation suggests this parameter may also have utility as an index of therapeutic efficacy.

## Background

NET disease is increasing in incidence and prevalence as attested to by national and internationally derived epidemiological data [1]. As a consequence of the increasing awareness of the disease and the introduction of novel efficacious therapeutic strategies (Everolimus, Sunitinib, Peptide Radio Receptor Therapy, surgical and radiofrequency ablative hepatic metastatic techniques), the clinical relevance of accurately determining the status of disease has become an issue of paramount importance. Although early diagnosis of NET disease remains a key challenge, a further critical emerging management issue is the limited ability to accurately gauge disease progress by imaging or biomarker assessment [2].

Failure to identify disease progress early and adjust therapy and the inability to delineate a lack of therapeutic efficacy and expeditiously introduce an alternative therapy are both equally deleterious to optimal management strategy and hence prejudicial to outcome. Thus, a critical limitation of outcome enhancement is reflective of three issues: 1) a paucity of specific targeted therapeutic agents and the inability to preemptively identify the molecular target; 2) imagery that is relatively insensitive due both to low discriminant index and the indolent nature of the disease and thirdly, a dearth of sensitive NET-specific biomarkers to identify alteration in disease status. In this respect, the currently used blood index, chromogranin A (CgA) is relatively non-specific, has low sensitivity, diverse assay interpretations of normality and defines a secretory product as opposed to specific indices of neuroendocrine tumor cell biology [3].

We have developed and published a blood based multigene (*n* = 51) transcript neuroendocrine specific index to identify NET disease status [4]. The sensitivity and specificity provide substantial information additive to current imaging techniques and plasma CgA levels in establishing alterations in disease status. This case illustrates the advantages inherent in utilizing multiple tumor-specific gene markers to identify early and specific changes in disease progression not detectable by standard imagery and biomarker analysis.

## Case presentation

A fifty-five year old male with a history of hypertension, hyperlipidemia and renal calculi presented in December 2001 with flushing and mildly elevated 24 hr urinary 5-hydroxyindole acetic acid (U-5HIAA) (“carcinoid syndrome”). A small bowel neuroendocrine tumor (NET:<2 cm) with two right lobe neuroendocrine liver metastases (NELMs) (2.8, 6.5 cm) was identified by OctreoScan® and MRI (CT identified one abnormality). A distal ileal resection with mesenteric lymphadenectomy, appendectomy and right lobe hepatic resection with cholecystectomy, was undertaken.

Histology (2001) indicated a Grade 2 NET, staging: T3, N2 (4/4), M1, G2, V1, R0. Ki-67 was not undertaken. His postoperative course was complicated by a right sub-diaphragmatic abscess, *Staphylococcus aureus* sepsis and was treated with antibiotics and percutaneous drainage. Annual follow-up, using CT, MRI, OctreoScan® and PET/CT, was instituted.

Initial progression free survival was three years. Thereafter, ^11^C-5HTP-PET/CT detected local mesenteric recurrence and re-resection of mesenteric lymph nodes was undertaken (March 2005, September 2006). Plasma chromogranin A (CgA) and U-5HIAAs were normal. A repeat ^11^C-5HTP-PET/CT (July 2007) identified no abnormal tracer accumulation, CgA was slightly elevated (23U/ml, *upper limit of normal = 19U/ml: DAKO ELISA Kit [K0025]* [5]) but U-5HIAA was normal. In April 2008, octreotide (20 mg) was empirically initiated (x1) but severe nausea and diarrhea precluded further therapy. CgA and U-5HIAA remained normal (June-July 2008). In October 2008, ^11^C-5HTP-PET-CT identified a solitary liver metastasis at the resection margin (right lobe); both CgA and U-5HIAA were normal, circulating 51 marker gene NET Index [4] were elevated. The metastasis was successfully percutaneously cryoablated (December 2008).

**Figure 1:**
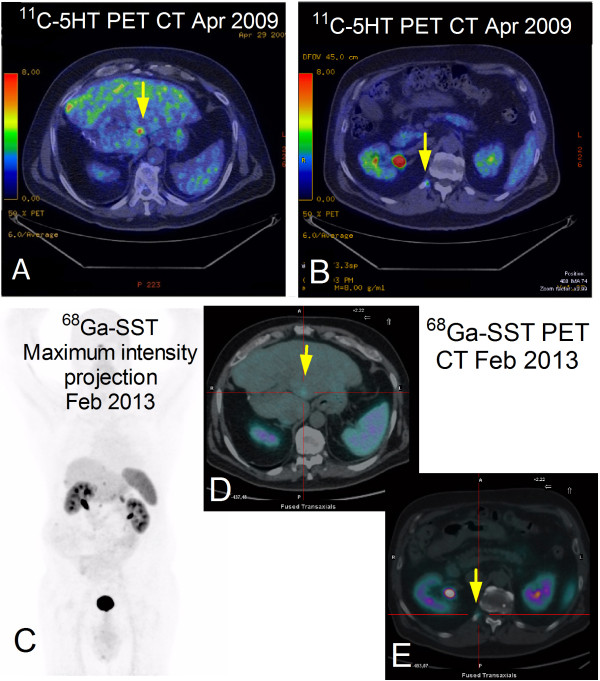
**Distinctive appearance at** ^**11**^ **C-HTP and** ^**68**^ **Ga-SST PET/CT of the liver (A and D) and bone metastases (B and E); no other lesions were detected at control PET scan performed with** ^**68**^ **Ga-SST (C).**

**Figure 2:**
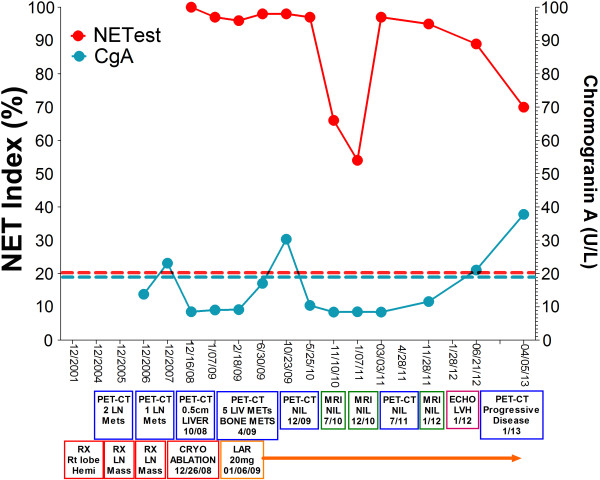
**Time line correlating plasma CgA measurements (DAKO ELISA, U/L) and the NET Index (%) (both y-axis) with the imaging and interventions (x-axis).**

In April 2009, ^11^C-5HTP-PET/CT demonstrated five small (<1 cm) NELMs and a rib lesion (Figure 1A-B*–yellow arrows*). CgA was elevated (30U/ml) as was the NET Index (Figure 2*–time line correlating plasma CgA with NET Index and imaging/interventions*). Retreatment with octreotide (20 mg/monthly) was initiated. After 8 months, lesions were no longer visible (PET/CT December 2009). No new lesions were evident on PET/CTs, MRIs and colonoscopy. In April 2011, CgA was normal. By June 2011, serotonin was slightly raised (402, ULN = 400U/L). PET/CT in June identified no abnormalities. The NET index was abnormal during this period.

A cardiac ECHO (January 2012) identified borderline LVH, and normal appearing tricuspid valves with trace insufficiency. In February 2013, ^68^Ga-DOTATOC-PET/CT identified hepatic recurrence (Figure 1C*–extent of disease*) in Segment IV, periphery of IV-V, and two lesions in Segment III (Figure 1D*–yellow arrow*). Right XII rib positivity was again noted (Figure 1E*–yellow arrow*). CgA levels were normal but the NET Index remained elevated. The patient currently exhibits stable residual NELM disease. A key management concern is the identification of progressive disease.

## Discussion

The detection of disease progression remains a key issue in the management of well-differentiated small bowel NETs. In most centers, plasma CgA is used in conjunction with a variety of imaging. Although widely used, CgA exhibits significant limitations in terms of sensitivity and specificity and is not elevated in a substantial percentage (15-47%) of NETs [3]. Imaging, both functional and topographical, is relatively insensitive in detecting alterations in indolent disease [6] and histopathological analysis of resected specimens indicates that imagery fails to detect ~50% of lesions [7]. Although the introduction of ^68^Ga-DOTA-PET and ^64^Cu-DOTATATE has amplified the ability to detect lesions, the former is not generally available and the latter is a research technique [8]. Strategies for early detection of disease recurrence or progression that inform timely treatment initiation are therefore suboptimal [9, 10].

### Imaging and biomarkers

Imaging (CT, MRI, OctreoScan®, ^68^Ga-DOTA-PET/CT) are considered preeminent modalities to assess disease stability and progression of NELMs [1]. There is, however, substantial variability in efficacy. The specificity for CT is as low as 22%, while both MRI and CT are negative in up to 50% of lesions [11]. The sensitivity (69-86%) of ^111^In-octreotide scintigraphy is lower than ^68^Ga-DOTA-PET/CT (^68^Ga-DOTATOC, -DOTANOC or -DOTATATE) [12] which exhibits the highest sensitivity and specificity for NELM (82–100%; 67–100%) and extra-hepatic metastasis (85–96%; 67–90%) detection. In addition, ^68^Ga-DOTA-PET/CT detects lesions not identified by CT and/or MRI in up to 67% of patients [6, 13]. ^18^F-DOPA-PET and ^11^C-5-HTP-PET have some utility in functionally active NETs but are not publically available. Furthermore, they are not theranostics and do not possess a therapeutic counterpart [14]. More recently, use of ^64^Cu-DOTATATE may surpass ^111^In and, theoretically, ^68^Ga in imaging sensitivity [8]. Irrespective, it is apparent that >50% of all NELMs will be under-staged (pathological analysis of surgical specimens) [7].

The use of individual peptides as biomarkers to identify early alteration in disease status has proved of limited value (e.g., pancreatic polypeptide) or amines (e.g., serotonin) although gastrin, glucagon and insulin are useful in specific NETs [15, 16]. Overall, the most widely used is CgA which broadly correlates with hepatic tumor burden and survival [17]. Elevations may be associated with tumor progression and in one report increased in 100% with progressive NELMs (disease relapse) [18]. In a retrospective analysis, a reduction of ≥80% was predictive of complete resolution of symptoms and disease stabilization [19]. In a separate study, CgA elevation was associated with residual disease [20]. Problems with CgA include no relationship to tumor grade (which is prognostic for survival), concerns regarding sensitivity and specificity, and the absence of any universally accepted assay methodology [3, 21]. The alternative, U5-HIAA, has limitations in terms of specificity and sensitivity [22, 23]. Nevertheless, a reduction of U5-HIAA levels ≥80% (or normalization) is reported as predictive of symptomatic relief, but not of disease progression [19].

Given the limitations of single agent biomarker analysis (CgA), we developed a multi-transcript (*n* = 51 gene) molecular signature for PCR-blood analysis based on specific neuroendocrine tumor cell transcripts identified by mathematical analysis of 15 NET tissue microarrays [4]. Gene co-expression network inferences and functional enrichment analyses of tumor tissue and peripheral blood NET transcriptomes (*n* = 22) identified 51 candidate genes. A test set of NETs (*n* = 130) was used to measure gene expression by hydrolysis-based qPCR and a tumor detection classifier was built using four learning algorithms (Support Vector Machine, Linear Discrimination Analysis, K-Nearest Neighbor and Naïve Bayes). This classification algorithm was validated in two independent NET sets (*n* = 115, *n* = 120) and exhibited a high sensitivity (85–98%), and specificity (93–97%) for NET detection including gastric, pancreatic and intestinal NETs. This significantly outperformed (ROC AUC: 0.95-0.98 vs. AUC: 0.64, *p* < 0.0001) CgA measurements [4]. Recently, this approach has been validated in a prospectively collected patient series [24]. To quantify data we developed a classification algorithm - NET Index (0 = no disease,100 = active disease) [25]. The index identifies progressive disease with a sensitivity and specificity of 91% respectively [25]. In this case study we evaluated the utility of blood CgA levels (ELISA) and the peripheral blood hydrolysis-based qPCR of the 51 marker genes (NET Index) derived from in using imaging as a baseline comparator.

#### 1) CgA levels

The first documented CgA measurement was made five years after initial diagnosis and was normal despite evidence of a mesenteric mass. Two years later, CgA levels remained normal despite a 0.5 cm NELM. CgA remained normal following cryoablation but became elevated after 2 months when bone and liver metastases were noted at PET-CT. Thereafter CgA levels normalized and remained within normal limits. Elevated CgA was only briefly detectable following cryotherapy when metastases were evident on imaging, but was normal when the hepatic metastatic burden was five lesions (>1 cm).

#### 2) NET index

Circulating tumor transcripts were measured from the same samples (collected from 2008) as CgA. PCR analysis and establishment of the NET index product can be made within 8 hours of blood collection. The NET Index was elevated (95–100) from initial visit (December 2008) when residual tumor was evident by imaging (CgA was normal). After cryotherapy, CgA levels decreased (30%) but blood transcripts remained elevated and were elevated two months prior to imaging detection of additional metastases (April 2009). The NET Index remained high despite initiation of octreotide (20 mg, January 2009) and only trended down in May and November 2010 when PET-CT identified no disease to be present. Lower levels appeared to correlate with efficacy of octreotide-therapy. Transcript levels remained low until January 2011 when progressive increases in the NET Index were noted. The highest NET Index (November 2011) was also concordant with the elevated serotonin; at this time, CgA levels were normal. The NET Index elevations preceded the ^68^Ga-PET CT identification of five NELMs (February 2013). It should be noted that both a functional PET/CT with ^11^C-5-HTP (July 2011) and an MRI (January 2012) failed to detect disease at these time points. It is likely that the five lesions noted in (2013) were too small to be detected by PET/CT and MRI (July 2011, January 2012 scans).

## Conclusions

This case report describes the limitations and discrepancies in assessing NET disease status by imaging and CgA. It provides preliminary information revealing the utility of a multi-transcript gene neuroendocrine tumor-selective panel.

Although CgA became transiently elevated following cryotherapy (evidence of NET destruction or surgical stress-related events), it has significant limitations for lesions ≤0.5 cm and can be normal despite the presence of somatostatin-avid lesions of ~1 cm [26]. Further difficulties are false positive elevations noted with concomitant proton pump inhibitor use and hypertension, cardiac disease and other endocrine pathology [3]. U-5HIAA can be falsely elevated by tryptophan-enriched foods and drugs but is elevated in ~88% of individuals with carcinoid syndrome (overall 10-15% of NETs) [22]. Twenty-four hour collection, storage and transportation render it inconvenient. The NET Index, in contrast, is not elevated by long-term PPI usage [4], cardiac disease or hypertension and was positive in all situations where imaging identified lesions (irrespective of size). Overall, the NET index was more sensitive than CgA in identifying neuroendocrine lesions and elevation was evident prior to image-based tumor confirmation in this patient. Measurement of a multi-transcript gene panel developed for gastroenteropancreatic NETs in blood provides a more sensitive and specific alternative to CgA in the diagnosis and management of NETs and with confirmation of these results in additional cases, demonstrate utility as an index of therapeutic efficacy.

### Consent

Written informed consent was obtained from the patient for publication of this case report and accompanying images.

## Abbreviations

5HTP: 5-hydroxytryptophan
CgA: Chromogranin A
CT: Computed tomography
DOPA: Dihydroxyphenylalanine
ECHO: Echocardiogram
GEP: Gastroenteropancreatic
MRI: Magnetic resonance imaging
NELM: Neuroendocrine liver metastasis
NET: Neuroendocrine tumor
PCR: Polymerase chain reaction
PET: Positron emission tomography
U-5HIAA: Urinary 5-hydroxyindole acetic acid.
